# Protein docking with predicted constraints

**DOI:** 10.1186/s13015-015-0036-6

**Published:** 2015-02-20

**Authors:** Ludwig Krippahl, Pedro Barahona

**Affiliations:** CENTRIA, Dept. Informática, FCT, UNL, Caparica, 2829-516 Portugal

**Keywords:** Docking, Constraints

## Abstract

This paper presents a constraint-based method for improving protein docking results. Efficient constraint propagation cuts over 95% of the search time for finding the configurations with the largest contact surface, provided a contact is specified between two amino acid residues. This makes it possible to scan a large number of potentially correct constraints, lowering the requirements for useful contact predictions. While other approaches are very dependent on accurate contact predictions, ours requires only that at least one correct contact be retained in a set of, for example, one hundred constraints to test. It is this feature that makes it feasible to use readily available sequence data to predict specific potential contacts. Although such prediction is too inaccurate for most purposes, we demonstrate with a Naïve Bayes Classifier that it is accurate enough to more than double the average number of acceptable models retained during the crucial filtering stage of protein docking when combined with our constrained docking algorithm. All software developed in this work is freely available as part of the Open Chemera Library.

**Electronic supplementary material:**

The online version of this article (doi:10.1186/s13015-015-0036-6) contains supplementary material, which is available to authorized users.

## Background

Proteins are large molecules formed by long chains of amino acid residues, often hundreds of residues long. The sequence of residues in each chain is determined by the DNA sequence of its corresponding gene, where each nucleotide triplet specifies which of 20 different amino acids will be covalently bound at that position of the chain, releasing one water molecule in the peptide bond reaction and leaving behind the amino acid residue, the remaining molecule, bound to the growing chain. Thousands of millions of years of evolution ensured that the proteins thus formed have a well defined structure and were selected for playing important roles in the biochemistry of the organism. In many cases, these roles involve specific interactions with other proteins and understanding which partners interact and the structure of the complexes formed by these protein interactions is a fundamental part not only of current fundamental research in molecular biology but also in applied fields such as drug design or metabolic engineering.

### Docking

Protein docking is the prediction of these protein-protein complexes from the known structures of the protein targets [1]. This is not an easy task, as demonstrated by the results of the Critical Assessment of PRediction of Interactions experiment, ongoing since 2001 [2]. According to the 2010 report for rounds 13-19, out of a total of 4420 submissions by 64 groups and 12 web servers, for six out of the thirteen target complexes there were no predictions considered highly accurate and only 16 models of at least acceptable quality in total, considering all submissions. Furthermore, one third of the participants failed to submit acceptable models for any target [3]. The more recent results, for rounds 20-27, was similar, with only four out of ten protein-protein docking targets with highly accurate results and 40% of the participants failing to submit even one model of acceptable accuracy [4].

Protein docking can be divided into two different tasks: filtering, wherein a large number of possible configurations is scanned and a small fraction is retained; and scoring, wherein each of the retained candidate models is examined in more detail so they can be ranked and, hopefully, the correct models identified [5]. These are sufficiently distinct problems that CAPRI even has a separate track for groups dealing solely with scoring. One possible way of improving protein docking results is to improve the scoring functions, and this is an important line of research. But another possibility is to improve filtering so that the scoring stage needs to rule out a smaller number of incorrect candidates in the search for the more accurate models. This is the goal of the work we describe here. From readily available sequence data, for each protein complex we obtain a set of potential contacts between specific amino acid residues. This set of contacts includes, with a good probability, at least one correct contact. By adequately processing the constraints derived from these contacts we can then quickly scan the whole set of potential contacts and increase the fraction of correct docking configurations in the set of candidate models.

### Contact prediction

At present, there are no reliable methods for accurately predicting specific residue contacts between proteins, though some success has been achieved in the prediction of interface regions, encompassing tens of amino acid residues on each partner, and these have been used to improve docking simulations. For example, PI-LZerD [6] uses predicted interface regions as an additional filter in the first docking stage by selecting models consistent with the predicted regions. However, this information is not used to actually guide the search for the initial set of models. Rather, it is added after the initial search, requiring that a very large number of models be kept initially (fifty thousand) to compensate this additional filtering. The authors of CPORT [7] report a similar problem, even though they were using interface predictions as restraints in HADDOCK [8], which uses ambiguous restraints as part of a cost function to minimize during search. The main problem remains that accurate models of the complex are often lost during the geometric search stage, a drawback that led us to consider an alternative approach to take advantage of the constraint programming approach in BiGGER [9,10]. By pruning the search space with constraints, it is more likely that correct models will be retained in the first filtering stage without requiring a very large set of candidate models. In previous papers we explored the possibility of improving docking simulations with geometric constraints [11], showed how propagation is implemented in BiGGER in order to allow pruning the search space with such constraints [9] and presented some preliminary work on how such constraints could be inferred from sequence data [12]. This paper focus on establishing a more solid foundation for this approach, outlining the procedure for processing sequences and obtaining the constraints and providing a practical tool that can be applied to real problems in the future.

### Our contribution

The goal of this work is to improve protein docking by restricting the search space with predicted inter-protein contacts. In particular, the main goal is to classify potential contacts in such a way that at least one correct contact can be kept in a small set of possibilities (e.g. 100 possible contacts). These possibilities are then scanned in a constrained docking simulation to obtain the most promising candidates using the BiGGER docking algorithm [9,10]. These predictions are derived from the analysis of Multiple Sequence Alignments (MSA) of homologous sequences in different organisms. The rationale is that, if homologs of both partners are present in different species, it is likely that they interact in a similar manner and that the residues contributing to this interaction will be under evolutionary constraints that can be detected in the sequence alignment. This can result, for example, in more conserved regions, correlated mutations due to coevolution, better residue complementarity and so forth. Since we try to predict contacts between specific amino acid residues, instead of predicting less specific interface regions, the prediction errors will be greater. However, specific contacts allow a much tighter pruning of the search space when processed with constraint programming, which can compensate for prediction errors by scanning a sufficiently large set of potential contacts.

Currently, one contribution from this work is the actual classifier and constrained docking software, which is open source and freely available. Although still not user-friendly, the software already allows the application of our classifier to predict the most likely contacts and use those predictions for constrained docking with the more recent version of BiGGER. However, a more important contribution is perhaps the demonstration of this framework for improving protein docking, which combines the prediction of specific contacts with a constraint programming approach, where the latter technique can help overcome the difficulties created by the large error margins in the predictions.

## Method

### Data preparation

From the Protein-Protein Docking Benchmark Version 4.0 [13] we selected all protein-protein complexes consisting of chains at least 50 amino acid residues long and whose structures had at most 10% unresolved residues. In addition, we excluded all antibody-antigen complexes because antibodies are generated by V(D)J recombination [14] and do not coevolve with antigens. We then searched for homologs of all sequences in the 50% identity clusters of the UniProt Reference Clusters database (UniRef50) [15], with the goal of obtaining a broad sample of sequence homologs. We queried UniRef50 to find matching clusters for each of our query sequences and then retrieved all sequences from each cluster. Preliminary results indicated that this is a better approach than a standard BLAST search [16] on individual sequences, which may not return enough sequences due to server-side limitations, and also better than PSI-BLAST [17] because PSI-BLAST finds more distant relatives through iterative queries against profiles determined by conserved regions. Although sequence conservation is one potentially useful feature, coevolution can also be indicative of a contact region and can only be detected in variable regions [18,19], so this bias towards conserved domains is not desirable. By using the UniRef50 database and retrieving the full clusters we can easily gather a large and unbiased sample of matching sequences using readily available services, which is important if our approach is to be of practical use to the community. We then matched and sorted all sequences obtained for each complex according to source organism, retaining only those sequences that could be matched to sequences for all other protein chains in the complex. The result was a pool of 103 complexes with at least 50 sequences for each chain. All sequence sets were aligned with Clustal Omega [20] (selecting a maximum of 2000 sequences, due to performance considerations, in the few cases where more were available).

These 103 complexes were randomly split into a training set of 75 complexes and a test set with the remaining 28 complexes. The training set was also randomly split into 5 partitions of 15 complexes each for estimating the classifier performances using five-fold cross validation. For each complex, we considered true contacts to be pairs of amino acid residues, one from each partner, with a distance no greater than 5Å from each other, as measured from the centres of non H atoms. This is the criterion used in the CAPRI programme for assessing protein interaction predictions [2]. It was also necessary to decide which residues would be considered as candidates for contact prediction. Only surface residues need to be considered, since buried residues cannot interact with the other partner, but it was necessary to decide how much surface area a residue would need exposed to count as a surface residue. The smaller this cutoff value, the greater the number of false contacts that will need to be classified. However, with larger cutoff values true contacts may be lost. Although we do not need many true contacts - in theory, identifying one would be enough to restrict the search space - we must not risk losing them all, so it was especially important to take into account those complexes with the smallest number of true contacts. Given these requirements we considered two indicators to minimize. One is the ratio of the average number of potential contacts to the minimum number of true contacts retained for any complex. The other is the fraction of lost true contacts in the complex with the smallest number of true contacts. Thus we selected a cutoff value of 38Å ^2^ because this is the value that minimizes the product of these two measures in our training set of 75 complexes. The exposed area estimates were computed using only heavy (non H) atoms, present in the PDB files. Up to a cutoff value of 38Å ^2^, the ratio between the total number of potential contacts and the minimum number of true contacts per complex decreased significantly from 2300:1 (for a surface exposure greater than 0Å ^2^) down to 780:1 while the minimum number of correct contacts kept per complex decreased only from 25 to 22. Beyond this cutoff value of 38Å ^2^ the reduction in the total number of potential contacts no longer compensates the loss of true contacts from the complexes with the smallest interfaces.

For all potential residue contacts, we computed a set of 21 base descriptors: maximum and minimum exposed surface for residues in the pair, both for the full residue and the side-chains; maximum and minimum substitution scores, using the Gonnet substitution matrix [21], for the substitution of each amino acid in the pair with all corresponding amino acids in the MSA and the same score relative to the average for the whole protein; interaction scores of the corresponding residues in the MSA averaged over all sequences, namely two volume normalized contact scores [22,23] and all atom and *α*C contact propensities [24]; maximum and minimum fraction of gaps in corresponding places in the MSA and maximum and minimum gap counts for each residue relative to the total gap counts for the MSA; SCOTCH interaction score [25] applied to the amino acid pair only, ignoring sequence neighbours). These 21 base descriptors were then aggregated over the spatial neighbourhood of each residue at the surface of the protein. This neighbourhood is defined as the specified residue plus all residues in the candidate set that are in contact with the specified residue. Thus, each of the initial 21 descriptors resulted in two additional scores: the average of the scores from all residues in one neighbourhood to the specific residue of the other partner (designated “all to one”), and the averages of all residues in one neighbourhood to all residues in the other neighbourhood (designated “all to all”). This resulted in an initial total of 63 descriptors to be used as features in the classifier. All values were scaled so as to range from -1 to 1 over the training set of 75 complexes for visual examination and comparison of features, with the same scaling factors were applied to the test set.

### Naive Bayes classifier

For this work, we chose to use a Naive Bayes Classifier (NBC). The NBC assumes that all features are conditionally independent given the class of the example, classifying each example according to the product of the probabilities of its features given each class and a probability distribution of each feature given each class. Although this is generally not true, it has the distinct advantage of allowing one to consider each feature independently and thus greatly simplifies the calculations required for feature selection. We chose to use an NBC because it generally performs well and because the independence assumption of the features is particularly suitable for using the classifier itself for feature selection, since the relevant probability distributions can be estimated beforehand, greatly speeding up the evaluation of feature combinations (see below). In an NBC, given the assumption that all features are conditionally independent given the class, the probability of vector ***x***, consisting of *d* features, belonging to a class *C*_*k*_ is proportional to:

(1) $$ p(C_{k} | \boldsymbol{x}) \propto p(C_{k}) \prod_{i=1}^{d} p(x_{i} | C_{k})  $$

Where *p*(*C*_*k*_) is the overall probability of belonging to class *C*_*k*_ and *p*(*x*_*i*_|*C*_*k*_) is the probability of feature *i* having value *x*_*i*_ if the class is *C*_*k*_[26]. To estimate the class conditional probability distributions we used the Equal Width Discretization (EWD) method, which consists of simply distributing the values of a continuous feature into *n* intervals of equal width. Despite its simplicity, EWD is reportedly a good method for NBC [27]. For each case, we chose *n* to be twice the cube root of the number of values, which is the Rice rule for choosing the number of bins in a histogram. This meant *n*≈200 for non-contacting residue pairs and *n*≈30 for contacting pairs, with a small variation depending on the training fold as the protein complexes have a different number of candidate pairs.

In practice, due to round-off errors, instead of the product of the conditional probabilities it is best to use the sum of the logarithms of these probabilities. Thus, for a generic problem with *N* classes and *d* features, the class is determined by

(2) $$ \begin{aligned} {} Class(\boldsymbol{x}) &= \text{arg\(_{k}\) max}\; Log \left(p^{\ast}(C_{k} | \boldsymbol{x}) \right)\\ {}\text{where}Log \left(p^{*} (C_{k} | \boldsymbol{x}) \right) &= Log \left(p(C_{k}) \right) + \sum\limits_{i=1}^{d} Log \left(p(x_{i} | C_{k}) \right) \end{aligned}  $$

Given that we have only two classes, being *C*_0_ the class of residue pairs that are not in contact and *C*_1_ the class of residue pairs in contact, we can use the NBC classification to score each ***x***:

(3) $$  S(\boldsymbol{x}) = Log \left(p^{\ast}(C_{1} | \boldsymbol{x}) \right) - Log \left(p^{\ast} (C_{0} | \boldsymbol{x}) \right)  $$

To evaluate the performance of each NBC (see section Feature selection) we used five-fold cross-validation over the training set of 75 complexes, measuring the R-precision of the NBC trained with each subset of features, averaged over the 75 complexes. R-precision is a standard measure used in document retrieval problems [28], and corresponds to the precision obtained in the first *R* results of a query, where *R* is the number of examples of the desired class. This is an appropriate measure because our contact prediction problem is analogous to a document retrieval problem in that we are interested in obtaining correct contacts close to the top of the ranking, thus reducing the number of potential pairs to test during the docking simulation before a good model of the complex is obtained. In our case, this means that, for each complex *j*, we ranked the potential contacts according to the *S* score in equation 3, counted the number of true contacts in the first *R*_*j*_ positions, where *R*_*j*_ is the total number of true contacts for that complex, and divided by *R*_*j*_. However, if there were no true contacts in the highest ranking *R*_*j*_ candidate pairs for complex *j*, then we modified the R-precision computation. In these cases, the standard R-precision measure simply assigns a value of 0 to the result. However, we were interested in measuring by how much the classifier failed to place a true contact in the highest *R*_*j*_. Thus, for complex *j*, we considered the value of R-precision of a given classifier to be:

(4) $$ {} Rprec(j) \,=\, \begin{cases} \frac{1}{R_{j}}\sum\limits_{k=1}^{R_{j}} isTrue(j,k), &\!\! \text{if} ~~highestTrue(j) \!\leq R_{j} \\ 1 - highestTrue(j)/R_{j}, &\!\! \text{if}~~ highestTrue(j) \!> R_{j} \end{cases}  $$

where *isTrue*(*j*,*k*) is 1 if the contact ranked in position *k* for complex *j* is a true contact, 0 otherwise, and *highestTrue*(*j*) is the position of the highest ranking true contact for complex *j*. The R-precision value assigned to each classifier was the average over all complexes classified.

### Feature selection

It would not be reasonable to assume that all descriptors would be useful features with which to classify the potential contacts, and in machine learning problems it is often necessary to select a subset of features from all descriptors available. To this end, we implemented a search algorithm [29] that first evaluates all features isolatedly, retains the best N, then tests each of those N combined with each of the other features, retains the best N pairs, and so forth, adding a new feature to each of the retained subsets at each iteration. Essentially, this is a forward sequential search but retaining N successors instead of just one at each iteration. In our case, the maximum R-precision value in cross-validation was obtained for a set of 20 out of the 63 original descriptors (see the Additional file 1 for a list of these descriptors). With more features the cross-validation score decreased consistently, so we stopped the search at 45 features. We used the 20 features that maximised the R-precision value to train our final classifier on the 75 complexes in the training set and then evaluate it on the 28 complexes of the test set.

## Results

On the test set, the final classifier ranked a median of 26.5 false positives higher than the first true contact. Considering that the median of the total number of contacts in the test set is 13770 and the median number of true contacts is 41, this means that the classifier is performing considerably better than a random guess (an average of 100 simulations using random scores gave a median ranking of 314 for the top ranking true contact). More relevant is the fact that the classifier ranks at least one true contact within the first 100 positions in 23 of the 28 test complexes, and one true contact in the highest 200 positions in 25 of the 28 test complexes. There is still room for improvement because, for the three complexes where the first true contact was ranked over 200, the rankings were 550, 888 and 1318, beyond what we consider to be useful. However, for any given complex, if we test the best 100 or 200 potential contacts it is likely we will find at least one true contact that will help guide the search for the right configuration. This is where the constraint programming approach comes into play. BiGGER can prune the search space using constraint propagation [9], making constrained docking very efficient. In the 28 test complexes, unconstrained docking runs took a total of 76 core hours at 2GHz for bound dockings, 89 hours for the unbound dockings. Due to the pruning of the search space, each constrained docking run is around 20 to 30 times faster than the unconstrained search, which allows us to screen the highest scoring 100 contacts predicted by the classifier in approximately ten to fiteen hours per complex, on average (279 core hours total for the bound dockings, 370 hours for the unbound dockings). Using multiple cores for a single docking run, easily available on modern personal computers, it is feasible to screen hundreds of potential contacts in a few hours with BiGGER. This is a typical time for protein docking runs and since the translational search is repeated for each of thousands of orientations of the ligand protein, parallelization has virtually no overhead. Unbound docking runs took, on average, one third longer than bound docking runs solely because the unbound structures were often larger than those found in the complex.

The remaining question was how beneficial this approach can be. To this end we compared constrained and unconstrained docking simulations on all 28 complexes of the test set. For the constrained simulations, we used the 100 highest ranking contact predictions. Thus, for 5 complexes there were no true contacts in the constraint set. The 28 complexes in the test set were modelled using both randomly oriented bound structures and unbound structures. Though in absolute values unbound docking results in a lower number of acceptable models, as expected due to the conformational differences between the unbound structures and the target complex, the relative gains due to using constraints are consistent across all cases.

For each docking run, we classified the models generated with and without constraints according to the ligand and interface root mean square deviations (rmsd). These are criteria used in the CAPRI programme [30], and consist in measuring the root of the mean of the squared distances between corresponding atom positions in two structures. The ligand rmsd score is measured by superimposing the structure of the larger partner (designated as the target) in the true, known, complex and the complex predicted by docking and then measuring the rmsd for the smaller of the two partners (called the ligand). This gives us a measure of the deviation in the position of the smaller partner, relative to the larger one, when compared with the true complex structure. The interface rmsd is the minimised rmsd score computed for all interface residues, defined as all residues from one partner that are within 5Å of any residue of the other partner, and measures the difference between the predicted and the actual interfaces. Following the criteria used in the CAPRI programme, we considered a model to be acceptable if the ligand rmsd is less than 10Å and the interface score is less than 4Å.

Table 1 shows both the bound and unbound docking results. The first column indicates the Protein Data Bank (PDB) identifier of the protein complex. The remaining columns group results according to the total number of models retained. Each cell shows the number of acceptable models obtained using constraints followed by a dash and the number of acceptable models obtained in the unconstrained docking. In the case of constrained docking, the total set of models retained was obtained by retaining the same number of models from each constraint, equal to 1% of the total. Thus, 5 models per constraint for the 500 models column, 10 for the 1000 models column, and so forth. The reason for sampling across constraints instead of aggregating all models is that incorrect models which happen to have a good surface contact can exclude acceptable models from the set of retained models. Sampling across constraints reduces this effect. Our results show that, on average, contact prediction significantly increases this number relative to unconstrained docking. The gain, measured by the ratio of acceptable models between constrained and unconstrained dockings, is, on average, 2.2 (*sigma*=0.2), and an analysis of variance for data with repeated measures (with and without constraints) indicates p values ranging from 0.003 to 0.00004, depending on the total number of models kept, so the results are all statistically significant. Despite this average effect, individual cases may be affected by other factors. The top five rows of Table 1 show cases where no correct constraint was identified in the set of 100 constraints used, so in theses cases constrained docking tends to give worse results, although some constraints may be incorrect by a sufficiently small margin to still allow acceptable models and even compensate the difficulties of unbound docking, as happens in complex 1akj. Even when correct constraints are predicted, these may not allow acceptable models given the conformation changes in unbound dockings (e.g. 7cei) or may result in a lower number of acceptable models if a significant number of unacceptable models have a higher surface contact score even when given a correct constraint (e.g 1i4d). In some fortuitous cases (e.g. 2o3b), the unbound structures happen to be less favourable to some high ranking incorrect models than to some acceptable models, leading to better results with unbound structures. In general, individual cases are influenced by many different factors but, on average, using predicted constraints according to the method we propose improves the number and fraction of acceptable models retained, a useful result to improve the chances of correctly identifying the structure of the complex.

**Table 1:** **This table shows the results for the 28 complexes in the test set**

	**Unbound**				**Bound**			
**PDB ID**	**500**	**1000**	**2000**	**5000**	**500**	**1000**	**2000**	**5000**
1gla	0 / 0	0 / 0	0 / 0	0 / 0	1 / 1	2 / 2	2 / 4	7 / 8
1y64	0 / 0	0 / 0	0 / 0	0 / 0	0 / 1	0 / 2	0 / 2	0 / 3
1akj	10 / 3	13 / 5	15 / 6	26 / 13	5 / 5	8 / 8	14 / 12	18 / 21
1s1q	0 / 0	0 / 0	0 / 0	0 / 0	4 / 12	5 / 16	6 / 30	8 / 41
3bp8	4 / 0	5 / 2	9 / 4	27 / 14	9 / 8	10 / 13	12 / 25	18 / 42
1pvh	0 / 0	0 / 0	0 / 0	0 / 0	2 / 1	2 / 1	3 / 2	5 / 3
2nz8	1 / 1	1 / 1	3 / 2	4 / 3	23 / 9	38 / 10	50 / 12	70 / 23
2j0t	2 / 2	3 / 2	7 / 3	11 / 7	10 / 5	12 / 5	20 / 8	30 / 20
7cei	0 / 1	0 / 1	0 / 1	0 / 4	5 / 1	6 / 1	7 / 1	7 / 1
1ijk	0 / 0	0 / 0	0 / 0	1 / 1	2 / 2	2 / 2	3 / 3	5 / 5
2o3b	0 / 0	0 / 0	0 / 0	10 / 4	0 / 0	0 / 0	0 / 0	1 / 1
1jwh	0 / 0	1 / 0	5 / 0	9 / 1	0 / 0	0 / 0	0 / 0	3 / 0
1i2m	0 / 0	0 / 0	0 / 0	3 / 0	1 / 1	3 / 1	6 / 3	12 / 6
2pcc	2 / 0	3 / 0	4 / 1	8 / 1	2 / 0	5 / 5	8 / 6	15 / 13
1h1v	0 / 0	0 / 0	0 / 0	0 / 0	2 / 2	3 / 3	5 / 6	10 / 8
1b6c	0 / 0	1 / 1	2 / 1	2 / 1	21 / 25	36 / 35	62 / 43	117 / 61
2hle	1 / 0	2 / 1	3 / 1	11 / 3	2 / 0	4 / 0	9 / 2	16 / 5
1bkd	0 / 0	0 / 0	0 / 0	0 / 0	19 / 6	35 / 9	67 / 15	106 / 20
1he1	11 / 6	18 / 12	30 / 17	53 / 34	32 / 3	42 / 5	77 / 9	140 / 17
1m10	16 / 5	19 / 5	27 / 11	54 / 17	36 / 9	54 / 14	75 / 17	106 / 22
1i4d	0 / 0	0 / 0	0 / 0	0 / 0	4 / 7	5 / 8	9 / 11	13 / 22
1azs	0 / 0	0 / 0	0 / 0	0 / 0	16 / 2	24 / 2	48 / 3	58 / 7
2sni	1 / 1	1 / 2	1 / 3	3 / 16	26 / 14	41 / 26	70 / 38	142 / 82
1gpw	0 / 1	2 / 1	2 / 3	13 / 10	25 / 14	42 / 22	73 / 26	132 / 41
1ofu	3 / 0	4 / 0	6 / 1	10 / 1	7 / 0	18 / 0	38 / 2	75 / 4
1z0k	1 / 1	3 / 1	16 / 4	39 / 20	36 / 17	58 / 27	92 / 43	179 / 85
1jzd	0 / 0	0 / 0	1 / 0	2 / 4	29 / 7	44 / 13	67 / 14	110 / 27
1fak	0 / 0	0 / 0	0 / 0	1 / 0	0 / 1	1 / 2	6 / 4	11 / 6
Av. gain	2,48	2,24	2,26	1,86	2,09	2,15	2,43	2,38

The top five complexes are those for which no true contact was ranked in the highest ranking 100 predicted contacts and had no correct constraints among the 100 used in docking. Unbound and bound docking runs are split into several columns for different numbers of models retained, and each cell shows the number of acceptable models using constraints and without constraints. The bottom row shows the average gain in acceptable models in each case, given by the total number of acceptable models using constraints divided by the total without constraints (including all complexes, even those without correct constraints).

### Conclusion

Our current results are quite promising given the significant increase in the number of good and acceptable complexes retained in the geometric search stage. Comparisons with other approaches to improving docking with contact predictions is not easy because these alternatives tend to strongly couple filtering and scoring and do not report on the specific effect of constraints in the filtering stage. For example, [6] report a maximum improvement of 17%, with bound docking simulations, on the number of complexes with acceptable predictions in the highest ranking models for the final score. It is not clear how to compare this result with our more than two-fold average increase in the number of acceptable models retained. However, experience with real docking applications shows that the scoring stage needs to be very flexible to account for the nature of each complex and the data available (see, for example, [31-36], but BiGGER has been used in over 70 published complex predictions) and so we should consider filtering and ranking the models as two distinct problems. In fact, filtering is a crucial stage because no scoring function at the ranking stage can help find an accurate model if no accurate models were retained during the filtering stage. This is also the reason why the CAPRI programme has independent tracks for scorers and predictors. Thus our focus here is on the filtering stage and on information that can be used to prune the search space. From our results we can conclude that efficient constraint propagation allows us to screen a large number of potentially correct constraints and thus make use of even very noisy data. In this work we presented a default scenario where the only source of constraints is contact prediction from sequence data, but often there is additional information that can provide more reliable constraints, further improving the results. We also present a framework for finding the appropriate constraints, although our current classifier probably can be still significantly improved. There are likely to be better descriptors than the ones we are currently using and, once a good set of descriptors are selected there are more powerful classification algorithms that we can use to find the right constraints (the main reason for using the Naïve Bayes classifier was for its efficiency in feature selection through model selection).

The source code for the software described in this work is part of the Open Chemera Library and is freely available at https://github.com/lkrippahl/Open-Chemera.

## Additional file

Additional file 1
**Training set, test set, and selected features.**
