# Supplementary material for: Protein docking with predicted constraints
Source: Algorithms Mol Biol. 2015 Feb 20;10:9. doi: 10.1186/s13015-015-0036-6 (PMC4340843; doi:10.1186/s13015-015-0036-6)
Supplement: Additional file 1 — Training set, test set, and selected features. [file 13015_2015_36_MOESM1_ESM.pdf]

Protein docking with predicted constraints (supplementary material)

Cross-validation of the Naïve Bayes classifier.

List of PDB identifiers for the 75 complexes used in the training set and the top ranking true contact predicted by the Naïve Bayes classifier using the selected features with five-fold cross validation. The value presented for each complex is the number of false contacts placed before the first true contact when that complex was evaluated in the validation fold (so when not used for training the classifier).

1ewy	26	1jk9	15	1mq8	40
1ira	2	1f51	38	1fq1	32
1oph	3	2c0l	133	1ib1	51
1fqj	37	1rv6	4	1e96	61
1mah	8	1ghq	111	1r6q	10
1ay7	3	2uuy	3	1n2c	98
1rlb	3	2j7p	209	1t6b	11
1xd3	2	1jiw	5	1r8s	3
1ibr	35	2z0e	0	1atn	51
2sic	25	1eaw	4	1zm4	37
1lfd	15	2o8v	0	1e6e	9
2abz	35	1gp2	0	1gcq	7
1kkl	16	2hrk	2	1kxp	17
2btf	29	1h9d	14	1pxv	131
1k5d	2	1buh	3	1syx	32
1ktz	4	2oza	3	1udi	50
1cgi	23	2oor	28	1wdw	17
1gxd	298	3cph	28	1z5y	13
1yvb	58	1qa9	74	1de4	134
1bvn	17	1ffw	5	1hcf	0
2hqs	64	1eer	42	1dfj	16
1f6m	1	2ayo	29	1xqs	11
1wq1	0	1us7	78	2cfh	1
1a2k	13	2a5t	17	1d6r	72
2ido	40	1grn	31	2mta	8

Additional data on the test set

This table shows additional details on the test set. The first column is the complex PDB identifier, followed by the total number of potential contacts considered for that complex, the number of true contacts and the number of false contacts that were ranked higher than the first true contact by the classifier. The times in the two following columns are for a single core, in hours, for the unconstrained docking and the 100 constraints of the constrained docking runs, for the bound dockings. The speedup is the unconstrained time divided by the average time to compute the docking results for a single constraint. Note that the speed-up tends to be greater for larger proteins, where docking takes longer. The total time for all 28 unconstrained dockings was 76.1 hours and 279.5 for constrained docking, which gives an average of 27.2 speed-up for the constrained docking, considering that each constrained docking run is in fact a run over 100 different docking constraints. The unbound docking runs took a total of 88.6 hours for the unconstrained dockings and 370.9 hours for the constrained dockings, for an average speedup of 23.1 for each constraint relative to the unconstrained docking. The differences are due to the unbound structures often being significantly larger than the structures determined in the complex.

				Bound		Unbound	
PDB id	Total Contacts	True Contacts	False Positives	Time (Unc.)	Time (100 Cs.)	Time (Unc.)	Time (100 Cs.)
1pvh	12474	36	0	1.6	7.3	2.0	20.6
2nz8	17072	59	52	3.3	12.2	3.6	26.3
2j0t	6622	33	28	1.0	7.5	1.4	12.5
7cei	4015	36	0	0.9	5.5	0.9	13.4
1ijk	13344	33	87	2.2	8.3	2.7	22.4
1gla	15744	27	550	2.1	9.7	2.0	14.7
2o3b	9184	36	3	1.3	9.3	2.0	16.6
1jwh	40136	44	23	5.0	14.4	5.1	28.9
1i2m	14196	64	23	2.7	10.4	3.5	21.4
2pcc	9280	25	54	1.2	8.1	1.5	17.2
1h1v	30770	55	45	3.8	13.8	9.3	32.0
1b6c	10944	37	10	2.2	9.3	2.6	20.0
2hle	9156	57	13	1.3	7.8	1.5	14.2
1y64	51506	61	1318	12.1	20.1	7.9	38.7
1bkd	24057	71	25	5.0	14.1	6.0	34.1
1he1	7885	52	3	1.1	5.9	1.4	14.8
1m10	13015	59	7	2.5	11.3	2.7	21.6
1i4d	19228	31	73	6.5	12.4	9.0	49.8
1azs	34225	36	19	3.9	14.5	4.5	21.9
1akj	24926	46	888	2.8	12.7	3.5	20.5
2sni	5640	41	51	0.7	5.1	0.9	12.4
1gpw	12423	37	20	1.8	10.2	2.0	17.2
1s1q	4230	36	118	0.6	4.7	0.7	11.6
1ofu	16800	45	23	2.7	11.9	3.2	22.1
3bp8	16873	37	193	3.7	7.3	1.9	19.8
1z0k	4464	41	29	0.6	5.1	0.7	10.2
1jzd	17100	50	4	1.5	7.9	2.3	19.2
1fak	19581	82	95	2.0	12.7	4.0	27.1

Selected features

The following 20 features were selected for use in the Naïve Bayes classifier, in decreasing order of importance:

Minimum Sidechain ASA
Maximum Sidechain ASA
Minimum Full ASA
Maximum Ful ASA
Contact Score Fraction(50full)AAcontacts.txt
Relative Contact Score Fraction(50full)AAcontacts.txt
Contact Score Fraction(50full)CAcontacts.txt
Relative Contact Score Fraction(50full)CAcontacts.txt
Contact Score Fraction(50full)EsqueVolNorm.txt
Minimum Full ASA (all to one)
Contact Score Fraction(50full)CAcontacts.txt (all to one)
Relative Contact Score Fraction(50full)CAcontacts.txt (all to one)
Relative Contact Score Fraction(50full)GlaserVolNorm.txt (all to one)
Min Gap Fraction(50full) (all to one)
Minimum Sidechain ASA (all to all)
Max Substitution Fraction(50full) (all to all)
Min Rel Substitution Fraction(50full) (all to all)
Contact Score Fraction(50full)AAcontacts.txt (all to all)
Max Relative Gap Fraction(50full) (all to all)
SCOTCH Score(50full) (all to all)

The minimum and maximum Accessible Surface Area values, computed both for the side chain and for the full amino acid residue, appear in the first four places, probably because interface residues generally are exposed to the solvent before the complex is formed. Thus the minimum and maximum values for each residue in the pair forming the potential contact are good initial discriminators. These values appear again further down in the list but computed between all neighbours (all to all) and between each residue in the pair and the other residue and all its neighbours (all to one). Next come contact propensities estimated from statistics on amino acid contacts in complexes (both the Glaser and Esque volume normalized statistics and the all atom and α-Carbon contact statistics, please see the main text for references). The relative contact score fractions are normalized by subtracting the expected value of randomizing the sequence correspondences. Finally, the selected features also include the substitution scores (using the Gonnet substitution matrix) and the fractions of gaps in the sequences for the corresponding residues. Since the Naïve Bayes classifier considers the distributions of all these features in the true and false contacts, features improve classification not merely for being informative in themselves but by adding information not provided by the other features. So, apart from the features in the top of the list, which were added first during the search, it is not possible to infer that each of the features is important in itself, merely that it helps to classify the contacts correctly when in conjunction with the remaining features.
